# Comparison of Preoperative Neutrophil-Lymphocyte and Platelet-Lymphocyte Ratios in Bladder Cancer Patients Undergoing Radical Cystectomy

**DOI:** 10.1155/2019/3628384

**Published:** 2019-10-02

**Authors:** Ruiliang Wang, Yang Yan, Shenghua Liu, Xudong Yao

**Affiliations:** Department of Urology, Shanghai Tenth People's Hospital, Tongji University, Shanghai 200072, China

## Abstract

**Introduction:**

Neutrophil-to-lymphocyte ratio (NLR) and platelet-to-lymphocyte ratio (PLR) have been proven to be significant prognostic factors in many cancers. We aimed to retrospectively investigate the prognostic value of NLR and PLR in patients with bladder cancer undergoing radical cystectomy.

**Materials and Methods:**

The study comprised patients from 2010 to 2018 who were diagnosed with bladder cancer and received radical cystectomy. Clinical and pathological parameters were collected. Receiver operating characteristic curves of NLR and PLR were plotted for overall survival (OS) and cancer-specific survival (CSS). The best cutoff value of NLR and PLR were determined using X-tile software. The prognostic value of NLR and PLR for OS and CSS was analyzed using the Kaplan–Meier method and Cox regression models.

**Results:**

A total of 223 patients were enrolled with a medium follow-up period of 57 months. Receiver operating characteristic curves showed that PLR was superior to NLR as a prognostic factor in patients with bladder cancer undergoing radical cystectomy. Univariate analysis revealed that NLR (*p*=0.032 and *p*=0.041) and PLR (*p*=0.003 and *p*=0.003) were significantly associated with both OS and CSS, respectively. Multivariate analysis identified only PLR as independent prognostic factors for OS (*p*=0.046) and CSS (*p*=0.039), respectively.

**Conclusions:**

The present findings suggested that compared with NLR, PLR was a superior prognostic factor of OS and CSS in bladder cancer patients indicated to radical cystectomy.

## 1. Introduction

Bladder cancer remains one of the most commonly diagnosed cancers in the urinary system. It is estimated that 80,470 new cases and 17,670 deaths from bladder cancer will occur in the United States in 2019 [1, 2]. Muscle-invasive bladder cancer, particularly, is featured with high relapse and mortality rate despite treatment effort over decades. Radical cystectomy has long been the golden standard in the treatment of muscle-invasive and high-risk non-muscle-invasive bladder cancer [3]. Preoperative clinicopathological factors, including tumor stage and nodal status, pathologic tumor grade, presence of lymphovascular invasion and carcinoma in situ, and the administration of chemotherapy, have been recognized to be linked with worse prognosis after radical cystectomy of bladder cancer [4–6]. However, they are not sufficient to guide clinical decision making. Therefore, other reliable pretreatment prognostic factors are urgent needed.

Host inflammatory responses play an important role in tumor development and progression. In recent years, inflammatory cells infiltrated in the tumor microenvironment and hematologic markers have been used in prognosis of various cancers, as well as urothelial carcinoma. Neutrophil-to-lymphocyte ratio (NLR) and platelet-to-lymphocyte ratio (PLR) are two main inflammatory- and immunologic-based scores identified as prognostic indicators in colorectal, gastric, lung, and ovarian cancers [7–10]. These two are inexpensive and convenient to apply in clinical practice. However, few studies had compared their prognostic value in bladder cancer patients simultaneously. In our study, we therefore conducted a retrospective study to evaluate the prognostic value of NLR and PLR in patients with bladder cancer indicated to radical cystectomy.

## 2. Materials and Methods

### 2.1. Study Patients and Design

Consecutive patients who were diagnosed with bladder cancer and underwent radical cystectomy from January 2010 to June 2018 in our hospital were enrolled in this study. Indications for radical cystectomy were strict to the guideline: muscle-invasive bladder tumor T2-T4a, N0-Nx, and M0 or high-risk recurrent non-muscle-invasive bladder tumors and BCG-refractory, BCG-relapsing, and BCG-unresponsive, and T1G3 tumors [11]. Patients receiving neoadjuvant chemotherapy or radiotherapy were excluded, since it might interfere with the white blood cell count. The medical records were reviewed retrospectively for clinical and pathological information playing an important role in prognosis of radical cystectomy [12], including age, gender, tumor stage, size, tumor grade, histology, lymphovascular invasion, hydronephrosis, history of adjuvant chemotherapy, and Charlson comorbidity index (CCI) score, measured using standardized International Classification of Diseases, Ninth Revision protocol. Cell counts from routine blood tests that were carried out within 3 days before surgery were obtained. Tumor grade was evaluated according to the 2004 WHO classification by a single group of pathology doctors in the Pathology Department in our institute. Pathological stage was reassigned according to the 2010 American Joint Committee on Cancer TNM Staging System. Lymphovascular invasion was defined as the presence of tumor cells in the lymphatic vessels and in vascular walls. The study was approved by the institutional review board of Tenth Hospital of Tongji University. Written informed consent was obtained from each patient. All data were anonymized before being used in this study.

### 2.2. Follow-Up

Patients were followed up every 3 months within the 2 years after radical cystectomy and then every 6 months annually. Follow-up consisted of medical history, physical examination, blood laboratory tests, and urine sedimentation. Imaging studies included computed tomography of chest abdomen and pelvis and bone scanning, which were obtained at 6 and 12 months postoperatively and annually after. Patients were followed till death or till the date of February 1, 2019. Endpoints were time to overall survival and cancer-specific survival. Overall survival (OS) was defined as the length of time from the start of cystectomy to the date of all-cause death. Cancer-specific survival (CSS) was defined as the length of time from the start of cystectomy to the date of death from bladder cancer.

### 2.3. Statistical Analysis

NLR was obtained by dividing absolute neutrophil count by absolute lymphocyte count, and PLR was obtained by dividing absolute platelet count by absolute lymphocyte count. X-tile software v3.6.1 (Yale University) was used to determine the optimal cutoff values of NLR and PLR [13]. Receiver operating characteristic (ROC) curves of NLR and PLR were plotted for the diagnosis of overall survival, and the area under the curve (AUC) was used to assess the classification performance of the model. Statistical significance of AUC was calculated by Delong's test [14]. The association between NLR, PLR, and the clinicopathological parameters was compared by Student's *t*-test for continuous variables and chi-square test for categorical variables. OS and CSS curves were drawn by the Kaplan–Meier method and evaluated by the log-rank test. Univariate and multivariate analyses were performed using the log-rank test and Cox proportional hazards regression models. Only factors determined to be significant according to the univariate analyses were subsequently included in the multivariate analyses (using forward: LR). Both NLR and PLR were included as the categorical variable. A value of *p* < 0.05 was considered to be statistically significant. All statistical analyses were performed using SPSS Version 22.0 (IBM Corporation, Armonk, NY, USA).

## 3. Results

A total of 224 patients received radical cystectomy in our hospital during the study period. One patient was excluded for receiving neoadjuvant chemotherapy. A total of 223 patients were enrolled in this study, including 146 males and 77 females, with the medium age of 67 years. Only 7 patients were lost during follow-up. The medium follow-up period was 57 months (range 8–116 months). A total of 80 patients died, and 69 of them died of bladder cancer during follow-up.

**Figure 1:**
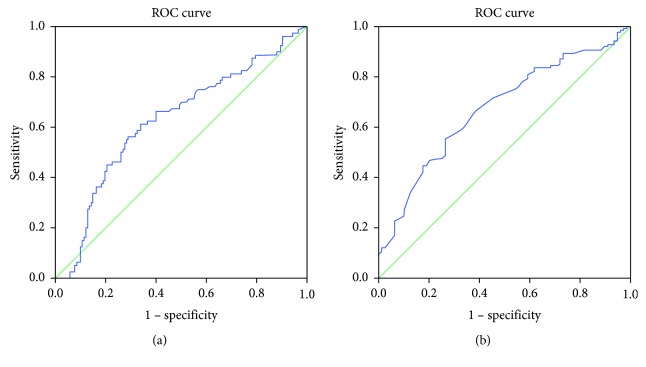
ROC curves of NLR (a) and PLR (b) for survival prediction.

Based on the results obtained from X-tile software, the best cutoff values were 4.1 for NLR and 164.7 for PLR. Based on the cutoff value of 4.1, 77 (34.5%) had elevated preoperative NLR. The area under the curve (AUC) of NLR was 0.617 (95% confidence interval: 0.538–0.695, *p*=0.004). When the cutoff was set as 164.7, 71 (31.8%) had rising preoperative PLR, with an AUC of 0.672 (95% confidence interval: 0.600–0.745, *p* < 0.001) (Figure 1). Our data showed that PLR was superior to NLR as a prognostic factor in patients with bladder cancer undergoing radical cystectomy (*p*=0.045, Delong's test).

According to the cutoff value, patients were divided into low- and high-NLR and low- and high-PLR groups. Comparison of patient's clinical and pathological variables according to different groups was shown in Table 1. Patients with elevated NLR were older at the time of radical cystectomy (*p*=0.043). The test on other variables, including gender, CCI score, T and N stage, tumor grade, lymphovascular invasion, carcinoma in situ, adjuvant chemotherapy, and preoperative hydronephrosis presented no significant differences among two groups.

**Figure 2:**
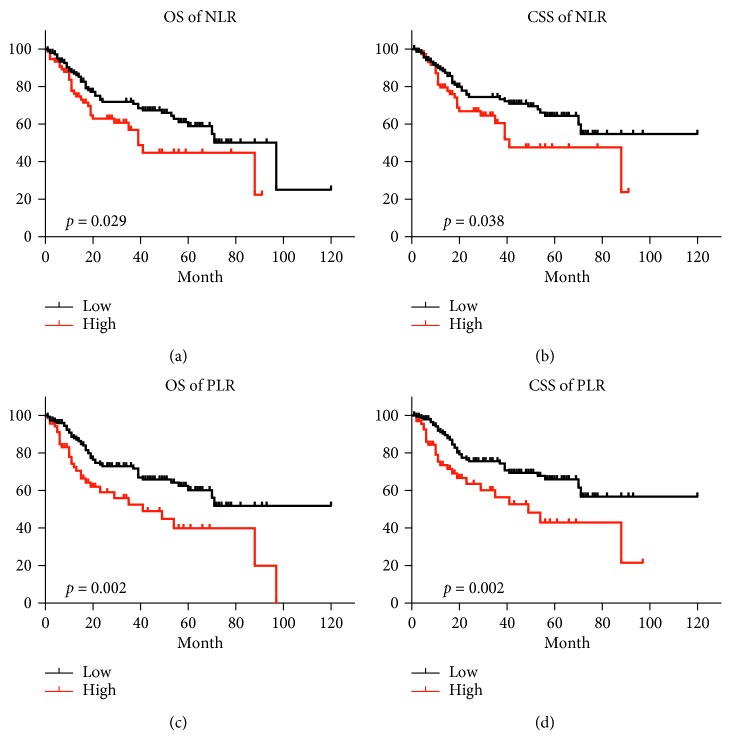
Kaplan–Meier curves for OS and CSS of patients with bladder cancer undergoing radical cystectomy. (a) OS curves of patients in the low NLR group (<4.1) vs the high NLR group (≥4.1), *p*=0.029. (b) CSS curves of patients in the low NLR group (<4.1) vs the high NLR group (≥4.1), *p*=0.038. (c) OS curves of patients in the low PLR group (<164.7) vs the high PLR group (≥164.7), *p*=0.002. (d) CSS curves of patients in the low PLR group (<164.7) vs the high PLR group (≥164.7), *p*=0.002.

Next, we observed patients in the low NLR or PLR group had a higher OS rate than those in the high groups, respectively (77.1% vs 55.5% for NLR, 76.3% vs 43.6% for PLR). The Kaplan–Meier analysis indicated that high NLR and PLR values were associated with shorter OS (*p*=0.029 and *p*=0.002, respectively). Similarly, the 5-year CSS rate was significantly higher in the low NLR or PLR group than those in the high group (79.9% vs 59.0% for NLR, 79.2% vs 46.9% for PLR). The Kaplan–Meier analysis indicated that high NLR and PLR values were associated with shorter CSS (*p*=0.038 and *p*=0.002, respectively) (Figure 2).

Univariate analysis showed that T stage (*p* < 0.001 and *p* < 0.001), N stage (*p* < 0.001 and *p* < 0.001), lymphovascular invasion (*p*=0.005 and *p*=0.002), preoperative hydronephrosis (*p* < 0.001 and *p* < 0.001), NLR (*p*=0.032 and *p*=0.041), and PLR (*p*=0.003 and *p*=0.003) were significantly associated with both OS and CSS. Multivariable analysis showed that PLR was an independent predictor of OS and CSS (*p*=0.046 and *p*=0.039), along with T stage (*p*=0.001 and *p* < 0.001) and N stage (*p*=0.046 and *p*=0.039) (Tables 2 and 3).

**Figure 3:**
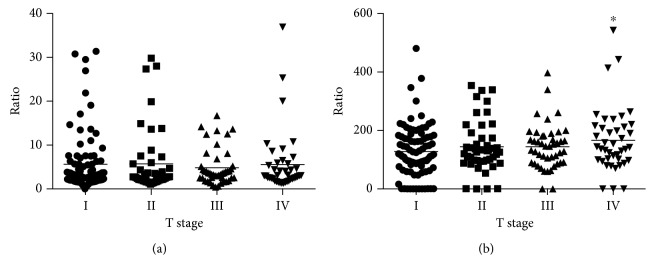
Association of NLR and PLR with TNM stage in patients undergoing radical cystectomy. (a) NLR. (b) PLR.

To validate the prognostic values of NLR and PLR in different T stages, a stratifying analysis was performed (Figure 3). The result revealed that the level of PLR had an increasing trend with the rising of T stage. Specially, the PLR level of stage IV patients was significantly higher compared with stage I patients (*p*=0.032). However, no significant difference was observed between the level of NLR and the T stage.

## 4. Discussion

Cancer and inflammation are greatly linked. Systemic inflammation and local immune response are two important parts contributing to the development and progression of malignancies. The cross-talk between two immune responses in cancer is complex [15]. Circulating immune cells with neutrophils being the most abundant, representing the systemic inflammation status, are likely to increase during cancer-mediated myelopoiesis. Tumor could also produce chemokines such as granulocyte colony-stimulating factor (G-CSF), interleukin-1, and interleukin-1 that lead to the elevated blood neutrophil [16]. Therefore, peripheral blood neutrophil-to-lymphocyte ratio is thought to reflect the development of tumor. Besides, immune cells could infiltrate and form part of tumor microenvironment. Low tumor-infiltrating lymphocytes has proved to be a worse prognostic biomarker and conducive to proliferation and metastasis of cancer [17]. In addition, neutrophils were also found infiltrating many types of tumors and playing both antitumor and protumor roles [18, 19]. However, elevated peripheral blood inflammatory cells do not necessarily mean increasing tumor-infiltrating ones. There was a negative correlation between NLR change and tumor-infiltrating lymphocyte change during chemotherapy in breast cancer [20].

The presence of platelets associated with cancer deposits has also been recognized. Tumor could activate platelets by generating platelet activators and mediators or providing direct contacting surface of tumor cell membrane. Conversely, platelets induce tumor angiogenesis by secreting proangiogenetic cytokines such as vascular endothelial growth factor (VEGF) and angiopoietin-1 [21]. Research showed preoperative blood platelet counts on its own could predict cancer mortality [22].

The prognostic value of NLR has been studied in bladder cancer. A meta-analysis study containing 11945 patients from 18 studies revealed NLR was significantly associated with decreased 3-year and 5-year overall survival and recurrence-free survival, but not with 10-year survival in primary bladder cancer patients who underwent radical cystectomy [23]. The cutoff value among different studies ranged between 2 and 5 [24]. Consistent with aforementioned results, we showed that NLR was a prognostic factor of bladder cancer in our cohort. With the cutoff value of 4.1, patients of high NLR had worse OS and CSS than those with low NLR.

PLR is another updated index that reflects the status of inflammation. High PLR level is linked to advanced tumor features and poor prognosis in breast, colorectal, and lung cancers [25–27]. However, the study of PLR on bladder cancer is scarce hitherto, and its prognostic role still remains controversial. A recent meta-analysis comprising only 3 low-quality studies showed elevated PLR was negatively related to the OS of different types of urological cancers, only except bladder cancer [28]. This might be due to the lack of enough, high-quality studies to draw a conclusive answer. Therefore, to further evaluate the role of PLR in more bladder cancer cohorts is warranted. Our study proved using the cutoff value of 164.7 that patients with high PLR had worse OS and CSS than those with low NLR. Moreover, a multivariate study showed PLR could be an independent risk factor of OS and CSS.

Although multiple clinical studies have explored the use of NLR or PLR, most studies only focused on a single indicator. In our study, we compared NLR and PLR simultaneously to evaluate the survival outcomes in bladder cancer. Both NLR and PLR had a good relationship with prognosis of bladder cancer undergoing cystectomy, whilst only PLR had statistical significance in multivariate analysis. The AUC of PLR was 0.672, statistically higher than 0.617 of NLR. This showed PLR was superior to NLR to predict the prognosis in our cohort. To our knowledge, only three studies have compared NLR and PLR together in bladder cancer undergoing radical cystectomy. However, the conclusions among studies were inconsistent. One study including 124 patients, supporting our views, showed that the high PLR group had worse OS, whilst NLR was not associated with oncology outcomes [29]. Another study including 144 patients, inconsistent with our result, showed that only NLR was statistically significant in multivariate analysis while PLR was not [30]. Compared with the above two, our study comprised a population of 223 patients, which was nearly twice as much of the population. The third case control study, including 127 bladder cancer patients and 162 healthy controls, showed both PLR and NLR could be significant independent predictors of urothelial bladder carcinoma [31]. This was partly because of different cutoff value sets and patient selection. They set a PLR value of 128.4 and included patients with more non-muscle-invasive patients who underwent transurethral resection of bladder tumor (TURBt). It is pivotal to set a proper cutoff value of NLR and PLR. In our study, we set the cutoff values using X-tile software, which could best differentiate two groups. Most of the previous studies only determined value empirically or median value [29].

There exist some limitations of our study. First, as our study is retrospective in nature and the data are from a single institution, our results have the potential to be biased in terms of the population choice. Second, all patients enrolled in our study were Chinese, so we cannot eliminate the influence of ethnic diversity. Third, the study sample is relatively small; thus, the significance of NLR and PLR needs to be verified in another large, prospective study. Fourth, other promising inflammatory markers, such as lymphocyte-to-monocyte ratio (LMR) and systematic inflammatory index (SII), were not simultaneously evaluated, which weaken the enrichment of the study.

## 5. Conclusions

Both NLR and PLR could be used as clinical indicators as their cheap and convenient features. The present findings suggested that compared with NLR, PLR was a superior prognostic factor of OS and CSS for bladder cancer patients indicated to radical cystectomy. Further study is warranted to verify the findings.

## Figures and Tables

**Table 1:** Relationships between clinicopathological parameters and NLR and PLR.

Variable	NLR < 4.1	NLR ≥ 4.1	*p* value	PLR < 164.7	PLR ≥ 164.7	*p* value
Age			0.043			0.146
Mean	65.57	68.52		65.89	68.07	
Median (range)	65 (29–87)	69 (32–87)		65 (29–87)	69 (32–87)	
Gender			0.216			0.513
Male	123	70		130	63	
Female	23	7		22	8	
CCI score	2.65 ± 0.88	2.68 ± 0.98	0.85	2.51 ± 0.88	2.60 ± 0.99	0.485
T			0.835			0.537
Tis, Ta, T1	57	31		61	27	
T2	33	14		34	13	
T3	29	16		31	14	
T4	25	16		24	17	
Tx	2	0		2	0	
N			0.34			0.095
N0	120	59		125	54	
N1-3	21	15		20	16	
Nx	5	3		7	1	
Grade			0.929			0.072
Low	8	4		11	1	
High	138	73		141	70	
LVI			0.333			0.892
Present	43	18		42	19	
Absent	103	59		110	52	
CIS			0.069			0.32
Present	10	1		6	5	
Absent	136	76		146	66	
Adjuvant chemotherapy			0.806			0.554
Present	19	11		19	11	
Absent	126	66		132	60	
Hydronephrosis			0.284			0.054
Present	34	23		33	24	
Absent	112	54		119	47	

CCI: Charlson Comorbidity index; LVI: lymphovascular invasion; CIS: carcinoma in situ; NLR: neutrophil-to-lymphocyte ratio; PLR: platelet-to-lymphocyte ratio.

**Table 2:** Univariate and multivariate analyses of factors associated with OS in bladder cancer patients undergoing radical cystectomy.

Variable	Univariate		Multivariate	
	HR (95% CI range)	*p* value	HR (95% CI range)	*p* value
Age	1.023 (1.001–1.046)	0.046		
Gender	1.055 (0.557–1.997)	0.869		
CCI	1.145 (0.904–1.451)	0.262		
Grade	1.853 (0.666–5.151)	0.237		
LVI	0.510 (0.320–0.813)	**0.005**		
Adjuvant chemotherapy	1.436 (0.828–2.489)	0.197		
Hydronephrosis	2.802 (1.792–4.381)	**<0.001**		
T stage	1.751 (1.440–2.130)	**<0.001**	1.531 (1.188–1.972)	**0.001**
N Stage	3.774 (2.303–6.186)	**<0.001**	2.888 (1.476–5.650)	**0.002**
NLR	1.643 (1.044–2.585)	**0.032**		
PLR	1.983 (1.262–3.116)	**0.003**	1.730 (1.010–2.964)	**0.046**

HR: hazard ratio; CCI: Charlson comorbidity index; LVI: lymphovascular invasion; CIS: carcinoma in situ; NLR: neutrophil-to-lymphocyte ratio; PLR: platelet-to-lymphocyte ratio.

**Table 3:** Univariate and multivariate analyses of factors associated with CSS in bladder cancer patients undergoing radical cystectomy.

Variable	Univariate		Multivariate	
	HR (95% CI range)	*p* value	HR (95% CI range)	*p* value
Age	1.023 (1.001–1.046)	0.046		
Gender	0.977 (0.484–1.971)	0.948		
CCI	1.120 (0.865–1.451)	0.391		
Grade	1.990 (0.622–6.361)	0.246		
LVI	0.454 (0.277–0.743)	**0.002**		
Adjuvant chemotherapy	1.569 (0.884–2.786)	0.124		
Hydronephrosis	2.993 (1.854–4.832)	**<0.001**		
T stage	1.949 (1.571–2.416)	**<0.001**	1.761 (1.336–2.321)	**<0.001**
N stage	4.187 (2.480–7.069)	**<0.001**	2.855 (1.401–5.819)	**0.004**
NLR	1.656 (1.020–2.687)	**0.041**		
PLR	2.083 (1.283–3.381)	**0.003**	1.761 (1.336–2.321)	**0.039**

HR: hazard ratio; CCI: Charlson comorbidity index; LVI: lymphovascular invasion; CIS: carcinoma in situ; NLR: neutrophil-to-lymphocyte ratio; PLR: platelet-to-lymphocyte ratio.

## Data Availability

The data used to support the findings of this study are restricted by the Ethics Board of Tenth Hospital of Tongji University in order to protect patient privacy. Data are available from Dr. Shenghua Liu (contact details: mobile: +8602166307458; e-mail: liushenghuafy@163.com) for researchers who meet the criteria for access to confidential data.

## References

[1] Siegel R. L., Miller K. D., Jemal A. (2018). Cancer statistics, 2018. *CA: A Cancer Journal for Clinicians*.

[2] Ploeg M., Aben K. K. H., Kiemeney L. A. (2009). The present and future burden of urinary bladder cancer in the world. *World Journal of Urology*.

[3] Alfred Witjes J., Lebret T., Compérat E. M. (2017). Updated 2016 EAU guidelines on muscle-invasive and metastatic bladder cancer. *European Urology*.

[4] Kluth L. A., Black P. C., Bochner B. H. (2015). Prognostic and prediction tools in bladder cancer: a comprehensive review of the literature. *European Urology*.

[5] Mari A., Kimura S., Foerster B. (2018). A systematic review and meta-analysis of lymphovascular invasion in patients treated with radical cystectomy for bladder cancer. *Urologic Oncology: Seminars and Original Investigations*.

[6] Soria F., Pisano F., Gontero P. (2018). Predictors of oncological outcomes in T1G3 patients treated with BCG who undergo radical cystectomy. *World Journal of Urology*.

[7] Haram A., Boland M. R., Kelly M. E., Bolger J. C., Waldron R. M., Kerin M. J. (2017). The prognostic value of neutrophil-to-lymphocyte ratio in colorectal cancer: a systematic review. *Journal of Surgical Oncology*.

[8] Miyamoto R., Inagawa S., Sano N., Tadano S., Adachi S., Yamamoto M. (2018). The neutrophil-to-lymphocyte ratio (NLR) predicts short-term and long-term outcomes in gastric cancer patients. *European Journal of Surgical Oncology*.

[9] Pavan A., Calvetti L., Dal Maso A. (2019). Peripheral blood markers identify risk of immune-related toxicity in advanced non-small cell lung cancer treated with immune-checkpoint inhibitors. *The Oncologist*.

[10] Prodromidou A., Andreakos P., Kazakos C., Vlachos D. E., Perrea D., Pergialiotis V. (2017). The diagnostic efficacy of platelet-to-lymphocyte ratio and neutrophil-to-lymphocyte ratio in ovarian cancer. *Inflammation Research*.

[11] Chang S. S., Bochner B. H., Chou R. (2017). Treatment of nonmetastatic muscle-invasive bladder cancer: American urological association/American society of clinical oncology/American society for radiation oncology/society of urologic oncology clinical practice guideline summary. *Journal of Oncology Practice*.

[12] Zhang L., Wu B., Zha Z., Qu W., Zhao H., Yuan J. (2019). Clinicopathological factors in bladder cancer for cancer-specific survival outcomes following radical cystectomy: a systematic review and meta-analysis. *BMC Cancer*.

[13] Camp R. L., Dolled-Filhart M., Rimm D. L. (2004). X-tile: a new bio-informatics tool for biomarker assessment and outcome-based cut-point optimization. *Clinical Cancer Research*.

[14] DeLong E. R., DeLong D. M., Clarke-Pearson D. L. (1988). Comparing the areas under two or more correlated receiver operating characteristic curves: a nonparametric approach. *Biometrics*.

[15] Diakos C. I., Charles K. A., McMillan D. C., Clarke S. J. (2014). Cancer-related inflammation and treatment effectiveness. *The Lancet Oncology*.

[16] Lechner M. G., Liebertz D. J., Epstein A. L. (2010). Characterization of cytokine-induced myeloid-derived suppressor cells from normal human peripheral blood mononuclear cells. *The Journal of Immunology*.

[17] Denkert C., von Minckwitz G., Darb-Esfahani S. (2018). Tumour-infiltrating lymphocytes and prognosis in different subtypes of breast cancer: a pooled analysis of 3771 patients treated with neoadjuvant therapy. *The Lancet Oncology*.

[18] Hurt B., Schulick R., Edil B., El Kasmi K. C., Barnett C. (2017). Cancer-promoting mechanisms of tumor-associated neutrophils. *The American Journal of Surgery*.

[19] Grecian R., Whyte M. K. B., Walmsley S. R. (2018). The role of neutrophils in cancer. *British Medical Bulletin*.

[20] Lee J., Kim D.-M., Lee A. (2019). Prognostic role and clinical association of tumor-infiltrating lymphocyte, programmed death ligand-1 expression with neutrophil-lymphocyte ratio in locally advanced triple-negative breast cancer. *Cancer Research and Treatment*.

[21] Marx S., Xiao Y., Baschin M. (2019). The role of platelets in cancer pathophysiology: focus on malignant glioma. *Cancers*.

[22] Vinholt P. J., Hvas A. M., Frederiksen H., Bathum L., Jørgensen M. K., Nybo M. (2016). Platelet count is associated with cardiovascular disease, cancer and mortality: a population-based cohort study. *Thrombosis Research*.

[23] Hu G., Xu F., Zhong K. (2018). The prognostic role of preoperative circulating neutrophil-lymphocyte ratio in primary bladder cancer patients undergoing radical cystectomy: a meta-analysis. *World Journal of Urology*.

[24] Marchioni M., Primiceri G., Ingrosso M. (2016). The clinical use of the neutrophil to lymphocyte ratio (NLR) in urothelial cancer: a systematic review. *Clinical Genitourinary Cancer*.

[25] Zhang M., Huang X.-z., Song Y.-x., Gao P., Sun J.-x., Wang Z.-n. (2017). High platelet-to-lymphocyte ratio predicts poor prognosis and clinicopathological characteristics in patients with breast cancer: a meta-analysis. *BioMed Research International*.

[26] Huang X.-z., Chen W.-j., Zhang X. (2017). An elevated platelet-to-lymphocyte ratio predicts poor prognosis and clinicopathological characteristics in patients with colorectal cancer: a meta-analysis. *Disease Markers*.

[27] Qiang G., Liang C., Xiao F. (2016). Prognostic significance of platelet-to-lymphocyte ratio in non-small-cell lung cancer: a meta-analysis. *OncoTargets and Therapy*.

[28] Li D.-Y., Hao X.-Y., Ma T.-M., Dai H.-X., Song Y.-S. (2017). The prognostic value of platelet-to-lymphocyte ratio in urological cancers: a meta-analysis. *Scientific Reports*.

[29] Zhang G.-M., Zhu Y., Luo L. (2015). Preoperative lymphocyte-monocyte and platelet-lymphocyte ratios as predictors of overall survival in patients with bladder cancer undergoing radical cystectomy. *Tumor Biology*.

[30] Rajwa P., Zyczkowski M., Paradysz A., Bujak K., Bryniarski P. (2018). Evaluation of the prognostic value of LMR, PLR, NLR, and dNLR in urothelial bladder cancer patients treated with radical cystectomy. *European Review for Medical and pharmacological Sciences*.

[31] Luo Y., Shi X., Li W. (2018). Evaluation of the clinical value of hematological parameters in patients with urothelial carcinoma of the bladder. *Medicine (Baltimore)*.

